# Human Case of Swine Influenza A (H1N1) Triple Reassortant Virus Infection, Wisconsin

**DOI:** 10.3201/eid1409.080305

**Published:** 2008-09

**Authors:** Alexandra P. Newman, Erik Reisdorf, Jeanne Beinemann, Timothy M. Uyeki, Amanda Balish, Bo Shu, Stephen Lindstrom, Jenna Achenbach, Catherine Smith, Jeffrey P. Davis

**Affiliations:** Centers for Disease Control and Prevention, Atlanta, Georgia, USA (A.P. Newman, T.M. Uyeki, A. Balish, B. Shu, S. Lindstrom, J. Achenbach, C. Smith); Wisconsin Division of Public Health, Madison, Wisconsin, USA (A.P. Newman, J.P. Davis); Wisconsin State Laboratory of Hygiene, Madison (E. Reisdorf); Sheboygan County Health and Human Services, Sheboygan, Wisconsin, USA (J. Beinemann); 1 Current affiliation: New York State Department of Health, Albany, New York, USA.

**Keywords:** influenza, swine influenza virus, zoonoses, dispatch

## Abstract

Zoonotic infections with swine influenza A viruses are reported sporadically. Triple reassortant swine influenza viruses have been isolated from pigs in the United States since 1998. We report a human case of upper respiratory illness associated with swine influenza A (H1N1) triple reassortant virus infection that occurred during 2005 following exposure to freshly killed pigs.

Human infections with swine influenza A viruses occur sporadically in the United States and Canada (*1*–*8*). Triple reassortant swine influenza viruses (containing genes derived from human, swine, and avian influenza A viruses) have been isolated from swine in the United States since 1998 (*9*,*10*), and human infections with swine reassortant viruses have been documented (*11*–*13*). We report a case of respiratory illness in an adolescent boy associated with swine influenza A (H1N1) triple reassortant virus infection.

## The Study

On December 7, 2005, a previously healthy 17-year-old boy with no history of recent travel became ill; symptoms were headache, rhinorrhea, low back pain, and cough without fever. He had received inactivated influenza vaccine administered intramuscularly on November 11, 2005. During an outpatient clinic visit on December 8, 2005, a nasal wash specimen was obtained and tested positive for influenza A by rapid influenza diagnostic test (BinaxNow A&B, Binax, Inc., Scarborough, ME, USA). Results of a chest radiograph were normal. The patient’s symptoms resolved on December 10, 2005. The specimen was sent to the Wisconsin State Laboratory of Hygiene (WSLH), and an influenza A virus was isolated by shell vial tissue cell culture (MDCK cells, WSLH, Madison, WI, USA). Real-time reverse transcription–PCR (rRT-PCR) was positive for influenza A virus but negative for human subtypes H1, H3, and Asian avian H5. At the Centers for Disease Control and Prevention (CDC), rRT-PCR testing of the shell vial viral culture material was positive for influenza A virus, but negative for human subtypes H1 and H3, as well as avian subtypes H5, H7, and H9. Complete genomic sequencing of the virus at CDC identified it as a swine influenza A (H1N1) triple reassortant virus, A/Wisconsin/87/2005 H1N1.

Investigation by the Wisconsin Division of Public Health and the Sheboygan County Division of Public Health showed that the patient had assisted his brother-in-law in butchering pigs at a custom slaughterhouse 3 days before illness onset. Thirty-one swine were delivered to the facility that morning by a distributor who had acquired the animals from multiple sources. None of the pigs appeared ill. The patient helped hold and abduct the forelimbs of 1 freshly killed pig while his brother-in-law eviscerated it. No facial or respiratory protection was worn during this procedure. A few chickens were housed at the slaughterhouse premises, but no poultry were slaughtered on site.

The patient denied any other contact with swine, poultry, or other animals during the 7 days before becoming ill. Eight days before illness onset, the patient’s father obtained a live chicken that was kept in the home for 1 day before it was sacrificed during a ritual ceremony. The patient was never within 10 feet of the chicken and did not attend the ceremony. None of the patient’s household members or any slaughterhouse employees reported illness during the 2 weeks before or after the patient became ill.

Paired serum specimens were obtained from the patient and 4 family members on December 13, 2005, and January 9, 2006. A single serum specimen was obtained from the patient’s brother-in-law on December 19, 2005. Serologic testing was conducted at CDC by a standard hemagglutinin inhibition (HI) antibody assay and reference antisera against influenza A (H1), A (H3), B, and swine A (H1N1) viruses A/swine/Wisconsin/238/97, A/swine/Wisconsin/NJ56371/99, A/swine/Minnesota/593/99, and A/Wisconsin/87/2005 (isolated from the patient). HI antibody testing was negative for influenza A (H1), A (H3), and swine subtype H1N1 on all serum specimens, but 1 family member had evidence of a 4-fold rise in HI antibody titer to influenza B/Hong Kong/330/2001 (B/Victoria/2/87 lineage) virus, which suggested an acute influenza B virus infection. All serum samples were also tested by microneutralization assay at CDC using the patient’s swine influenza A (H1N1) virus isolate and a human influenza A virus (A/New Caledonia/20/99 H1N1). The patient’s serum specimens demonstrated a 2-fold increase in neutralizing antibody titer against swine influenza A/Wisconsin/87/2005 subtype H1N1 virus, but the level of neutralizing antibodies to A/New Caledonia/20/99 (H1N1) virus was unchanged in acute- and convalescent-phase serum specimens, which is consistent with his history of influenza vaccination in mid-November 2005.

## Conclusions

We report a human case of swine influenza A (H1N1) triple reassortant virus infection in the United States. The case-patient experienced a mild and acute respiratory illness and recovered fully. Swine influenza A/Wisconsin/87/2005 (H1N1) virus was isolated from an upper respiratory specimen obtained from the patient, and serologic testing suggested, but was not diagnostic of, an immune response to acute infection. Epidemiologic investigation showed the patient had direct and close exposure to freshly killed pigs and their organs while assisting his brother-in-law in butchering them. Although the pigs did not appear ill, the most plausible source of the patient’s swine influenza A virus infection was respiratory secretions of freshly killed pigs.

Surveillance data suggest that triple reassortant subtype H1N1 viruses are the predominant genotype of subtype H1N1 viruses in North American pigs (*14*). Persons having direct contact with swine are at greatest risk of infection with swine influenza viruses (*4*–*7*,*13*), but such contact is not documented in all cases (*7*). Human-to-human transmission of swine influenza virus is rare, but evidence suggests that it has occurred (*1*–*3*,*7*,*8*,*13*). Human illness caused by infection with swine influenza viruses is often indistinguishable clinically from infections caused by other influenza viruses (*1*,*7*,*12*,*13*); complications, including pneumonia and death, have been documented (*3*,*4*,*7*). Asymptomatic infections in humans caused by swine influenza viruses may occur (*5*,*8*,*13*) and therefore, the true frequency of swine-to-human influenza virus transmission is unknown (*3*,*5*–*7*).

We were limited in assessing other possible swine influenza A (H1N1) virus infections and in confirming swine influenza in the pigs. Pigs delivered to the slaughterhouse the day of the patient’s exposure originated from multiple farms, but specimens were unavailable for testing due to delays during animal traceback. Our findings suggest that microneutralization assay may be more sensitive than a standard HI assay in detecting human antibodies to swine influenza A viruses. Primers and probes for detection of human influenza A viral RNA by rRT-PCR identified a nonhuman influenza A virus, triggering further analyses that specifically identified the virus. We could not confirm whether the patient’s influenza vaccination and high levels of vaccine-derived subtype H1N1 neutralizing antibody influenced his relatively mild clinical course of illness. The patient did not have a 4-fold increase in neutralizing antibody titer to the swine influenza A (H1N1) virus isolated from his respiratory specimens, which would be more suggestive of acute infection. Further studies are needed to understand the human immune response to infection caused by swine influenza A viruses and to interpret serologic test results.

Human infections with novel influenza A subtype viruses are now nationally notifiable in the United States. Clinicians should inquire about exposure to animals (including pigs) and visits to petting zoos and county fairs when evaluating patients with unexplained influenza-like illnesses (*15*). Ideally, joint animal health and public health investigations should be conducted promptly to identify and control the source of swine influenza. Investigations should attempt to specifically identify the virus in animals and persons; define the scope and clinical spectrum of human illnesses, including appropriately timed collection of serum specimens from ill persons and exposed individuals; determine risk factors for human infection; and assess the potential for human-to-human transmission of swine influenza A viruses.
